# The Role of the Microbiome in the Resolution of Infection-Induced Inflammation

**DOI:** 10.1021/acsinfecdis.5c00668

**Published:** 2025-10-24

**Authors:** Kerry McGowen, Junhee Lee, Virginia A. Pedicord

**Affiliations:** 1 2152 Cambridge Institute of Therapeutic Immunology and Infectious Disease, Jeffrey Cheah Biomedical Centre, Cambridge Biomedical Campus, Cambridge CB2 0AW, U.K.; 2 Department of Medicine, University of Cambridge School of Clinical Medicine, Cambridge Biomedical Campus, Cambridge CB2 0SP, U.K.

**Keywords:** inflammation resolution, infection, microbiome, tissue repair, immunity

## Abstract

The host microbiome plays a crucial protective role against pathogenic infections, not only through direct competition with invading pathogens but also by coordinating host antimicrobial and barrier functions and educating immune cells. While essential for pathogen clearance, unchecked, prolonged, or excessive inflammation from host immune responses can paradoxically lead to serious consequences for the host including the development of chronic inflammatory and autoimmune diseases. Recent research highlights how microbiome disruptions can exacerbate infection-associated inflammation and pathology. Even with established links among microbes, inflammation, and infection susceptibility, a comprehensive understanding of the cellular and molecular mechanisms connecting the microbiome’s role in resolving infection-associated inflammation remains largely undefined. This review discusses our current understanding of the microbiome’s contribution to resolving inflammation and tissue damage postinfection and its potential impact on therapeutic approaches for alleviating infection-induced inflammatory diseases.

## Graphical abstract

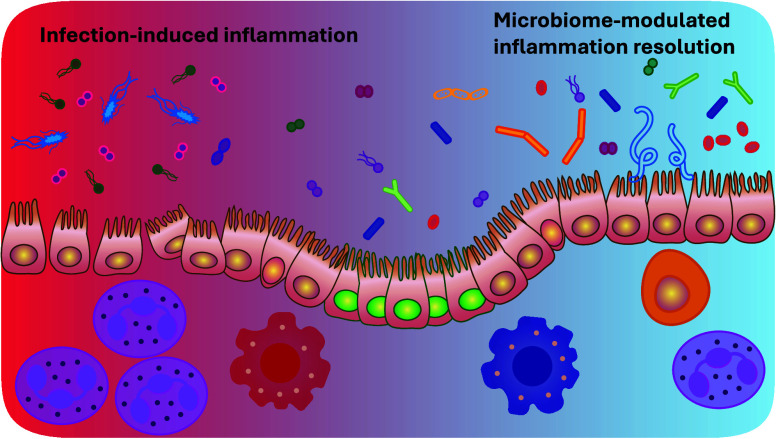

## Introduction

For more than one hundred years, resident gut microbes have been shown to play protective roles against pathogenic infections.^1^ In addition to directly competing with invading pathogens, the gut microbiota orchestrates a number of host responses involved in antimicrobial and barrier functions, including epithelial cell production of mucus and antimicrobial peptides as well as the activation and education of local immune cells.^2–4^ Together, these microbiota-dependent responses create environments that are often remarkably resistant to pathogenic infection and support the clearance of any invaders that manage to breach these resistance mechanisms. However, the pathology caused by infectious agents is often not confined to direct actions of the microbial pathogen.

The same immune responses that are required for pathogen clearance can cause inflammation and tissue damage that require active resolution to return to homeostasis. Previous work has shown that cues from resident microbes are important for shaping both inflammatory and anti-inflammatory immune responses as well as pathways for nonhematopoietic cell proliferation and tissue repair.^5–8^ More recent studies have also connected alterations in the gut microbiota to the development of inflammation and increased pathology in response to pathogenic infection. For example, antibiotic-induced microbiota disruption was recently shown to decrease type 2 innate lymphoid cells (ILC2) and their production of amphiregulin (Areg), an important regulator of intestinal tissue regeneration and repair.^7^ Depletion of ILC2 and Areg by antibiotics led to increased susceptibility to *Clostridioides difficile* infection-mediated mortality, while administration of exogenous Areg reduced *C. difficile*-associated pathology.

Although the connections between resident microbes, inflammation, and infection susceptibility are increasingly well-established, the cellular and molecular mechanisms that connect the microbiota to the resolution of inflammation, and specifically infection-associated inflammation, remain incompletely understood. Through understanding key contributions of commensal microbes to pro-resolving immune and tissue repair programs, better strategies can be devised for mitigating or treating infection-induced damage and inflammatory disease. In this review, we will examine what is currently known about how the microbiota contributes to minimizing and resolving inflammation and tissue damage in the wake of pathogenic infections.

## Commensal Microbes Can Protect against Pathogenic Infection and Promote Pathogen Clearance

For more than a century, scientists and physicians have accumulated direct evidence that resident gut microbes can affect pathogenic infection susceptibility and severity. In 1917, German physician Alfred Nissle observed that one World War I soldier in the Balkans did not fall ill from dysentery, unlike the rest of the soldiers. He isolated a strain of *Escherichia coli* from the soldier’s stool, which came to be known as the *E. coli* Nissle strain, and later showed this strain of bacteria to be protective against *Shigella*. Although the mechanism of infection protection by the Nissle strain has largely been characterized as colonization resistance mediated by niche competition with invading pathogens (Figure 1),^9^ recent studies have expanded the investigation of its beneficial effects. Beyond niche competition, outer membrane vesicles from *E. coli* Nissle have been observed to modulate immune cell activity,^10^ and current studies are genetically engineering this strain to develop therapeutic protein matrices that improve intestinal epithelial barrier integrity.^11^ These innovations have the potential to extend the probiotic applications for *E. coli* Nissle to numerous diseases, including inflammatory diseases like ulcerative colitis.^12^

**Figure 1:**
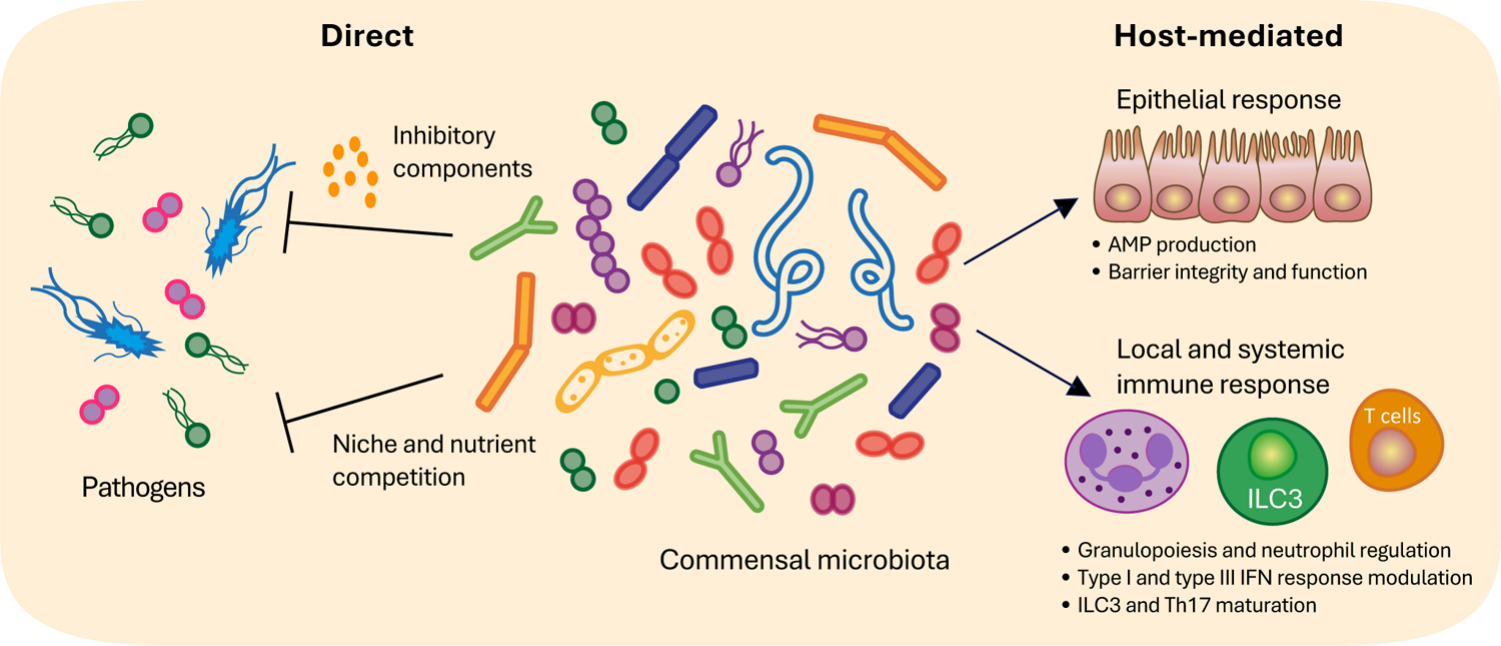
Commensal-mediated protection against pathogens. Commensal bacteria have been shown to provide resistance against pathogenic infection through both direct and indirect mechanisms. Species like *E. coli* Nissle have been shown to directly inhibit colonization of pathogens through niche competition and production of inhibitory bacterial molecules. Other commensals, such as *E. faecium*, have been shown to provide protection through the activation of host pathways, promoting intestinal stem cell survival and antimicrobial peptide production. Commensals have also been shown to promote both local and systemic innate and adaptive immune responses that enhance host protection against pathogenic bacteria and viruses.

Although *E. coli* was among the first members of the human gut microbiota to be characterized for its protective effects against infection-mediated pathology, it is rarely highly abundant within a diverse gut ecosystem, and numerous other species of gut bacteria and their metabolites have been shown to modulate host responses to infection. We have previously demonstrated the protective effects of a commensal strain of *Enterococcus faecium* on the antimicrobial peptide production and barrier function of intestinal epithelial cells (Figure 1).^13^ This was mediated by *E. faecium*’s secreted peptidoglycan hydrolase and the resulting production of muropeptides that robustly activate host protective pathways via the innate immune receptor NOD2.^14^ NOD2 stimulation by commensal gut bacteria has been shown to promote intestinal stem cell survival and protection against reactive oxygen species (ROS) mediated cytotoxicity.^15^ Recently, we also identified another gut species, *Enterocloster clostridioformis*, that decreases *Salmonella*-mediated pathology without competing for its metabolic niche or decreasing pathogen colonization (Figure 1).^16^ Collectively, these and numerous other studies support the notion that specific resident gut microbes can modulate infection-associated pathology through mechanisms that are distinct from their contributions to colonization resistance.

In addition to protection against pathogenic infection, the gut microbiome shapes both local and systemic innate and adaptive immune responses that support pathogen clearance (Figure 1). For example, microbiota-enhanced granulopoiesis and neutrophil regulation have been shown to contribute to controlling *Listeria* and pathogenic *E. coli* and *Klebsiella* infections, which were impaired in germ-free (GF) and antibiotic-treated mice.^17,18^ The maturation of type 3 innate lymphoid cell (ILC3) and Th17 responses has also been mechanistically linked to the presence of several adherent commensal gut microbes and retinoic acid production. These responses are essential for the control and clearance of bacterial and fungal pathogens both in the gut and at distal sites of infection.^19,20^ In addition, commensal microbiota can also modulate antiviral activity through interferon (IFN) responses. For example, in norovirus infection models, resident microbes can provide resistance in the proximal gut by priming type III IFN responses via the generation of secondary bile acids.^21,22^ Two recent studies have also demonstrated a crucial role for commensal bacteria-induced type I IFN in responses to viral infection with vesicular stomatitis virus or influenza^23^ and the ability of dendritic cells (DCs) to prime virus-specific T cell responses.^24^ However, another recent study revealed that manipulating the microbiota for the enhancement of T cell-mediated viral clearance in systemic LCMV infection also led to increased LCMV-associated pathology,^25^ demonstrating the potential for microbiota-modulated pathogen-clearing immune responses to result in collateral damage to the host.

## The Immune Response to Infection Can Cause Acute and Chronic Inflammation and Tissue Damage

Although research has extensively focused on the exclusion or clearance of pathogens and how commensal microbes support these processes, for some infections, direct tissue damage and pathology caused by the microbial pathogen itself are relatively minimal. Instead, the immune response and corresponding inflammation are responsible for a majority of infection-associated morbidity. Triggering of acute inflammatory immune responses via pathogen-associated molecular pattern (PAMP) engagement of innate immune receptors typically precedes and potentially prevents the tissue damage caused by an infectious agent.^26^ However, this host-preemptive-strike strategy inherently involves a risk that immune activation levels will exceed those required for the specific microbial threat. To combat this risk, a multitude of regulatory mechanisms exist to halt acute inflammation, but some degree of damage is likely unavoidable. A classic example is the induction of sepsis by a bacterial endotoxin. In sepsis, even minute amounts of endotoxin can trigger rapid immune dysregulation, including neutrophil activation and cytokine storm, that can lead to life-threatening organ dysfunction.^27^ In a separate example, several studies have characterized the role of bacterial T cell superantigens in *Staphylococcus aureus* pathogenesis, where massive T cell activation contributes to diseases such as toxic shock syndrome and pneumonia in the wake of *S. aureus* infection.^28,29^

With the potential disconnect between inflammation and direct pathogen interactions, the possibility arises for pathology to continue long after pathogen clearance. Indeed, in addition to acute damage and inflammation, certain infections have been shown to kickstart numerous chronic inflammatory and autoimmune conditions. For example, group A *Streptococcus* infection has long been directly linked to rheumatic fever and other autoimmune disorders,^30^ and *Helicobacter pylori* infection can lead to residual inflammation and gastritis well after successful eradication of the pathogen by antibiotics.^31^ In the latter example, *H. pylori* can colonize somewhat asymptomatically for decades, without causing severe acute inflammation or significant tissue damage; however, a combination of host immune activation and dysbiosis of the gastric microbiota has been shown to contribute to the development of chronic inflammation and subsequent gastric cancer associated with *H. pylori* infection.^32^ Other types of chronic illness have also been linked to prior infections. For instance, postinfectious irritable bowel syndrome (PI-IBS), a subset of IBS, is also rooted in enteric bacterial infection, and numerous pathogen-induced immune alterations in mast cells, T cells, macrophages, and pro-inflammatory cytokines have been linked to the disease.^33,34^ Together, these findings suggest that effective inflammation resolution is likely required to prevent chronic inflammation postinfection.

## Inflammation Resolution Is a Regulated and Active Process

Given the potential role of unresolved inflammation in chronic inflammatory disorders, it is important to understand the regulation of inflammation resolution and how it can mitigate the risk of long-term damage from infections. Inflammation resolution involves an active, tightly regulated series of biochemical and cellular processes.^35–37^ Briefly, the initiation of resolution involves the removal of inflammatory stimuli, which, in the case of infections, entails the removal of the pathogen. Then, pro-inflammatory signals and mediators must be dampened and then catabolized to prevent excessive damage from a prolonged pro-inflammatory state. This is followed by several key steps of inflammation resolution, including (1) termination of neutrophil recruitment, (2) induction of neutrophil apoptosis, (3) neutrophil removal through macrophage engulfment or efferocytosis, and (4) macrophage reprogramming from pro-inflammatory (M1-like) to pro-resolving (M2-like) phenotypes, directly induced by processes like efferocytosis of polymorphonuclear leukocytes (PMNs), leading to the release of anti-inflammatory cytokines such as IL-10 and TGF-β (Figure 2).^38–40^ Broadly, this process relies on the biosynthesis and control of specialized pro-resolving lipid or protein mediators (SPMs)^41^ that signal through G-protein coupled receptors on many different cell types to stimulate the cessation of pro-inflammatory pathways and initiate tissue repair (Figure 2).^41^ The most studied SPMs, resolvins, maresins, protectins, and lipoxins, are primarily produced by neutrophils, macrophages, and monocytes, with only a few types of SPMs found to be produced by T cells.^42^

**Figure 2:**
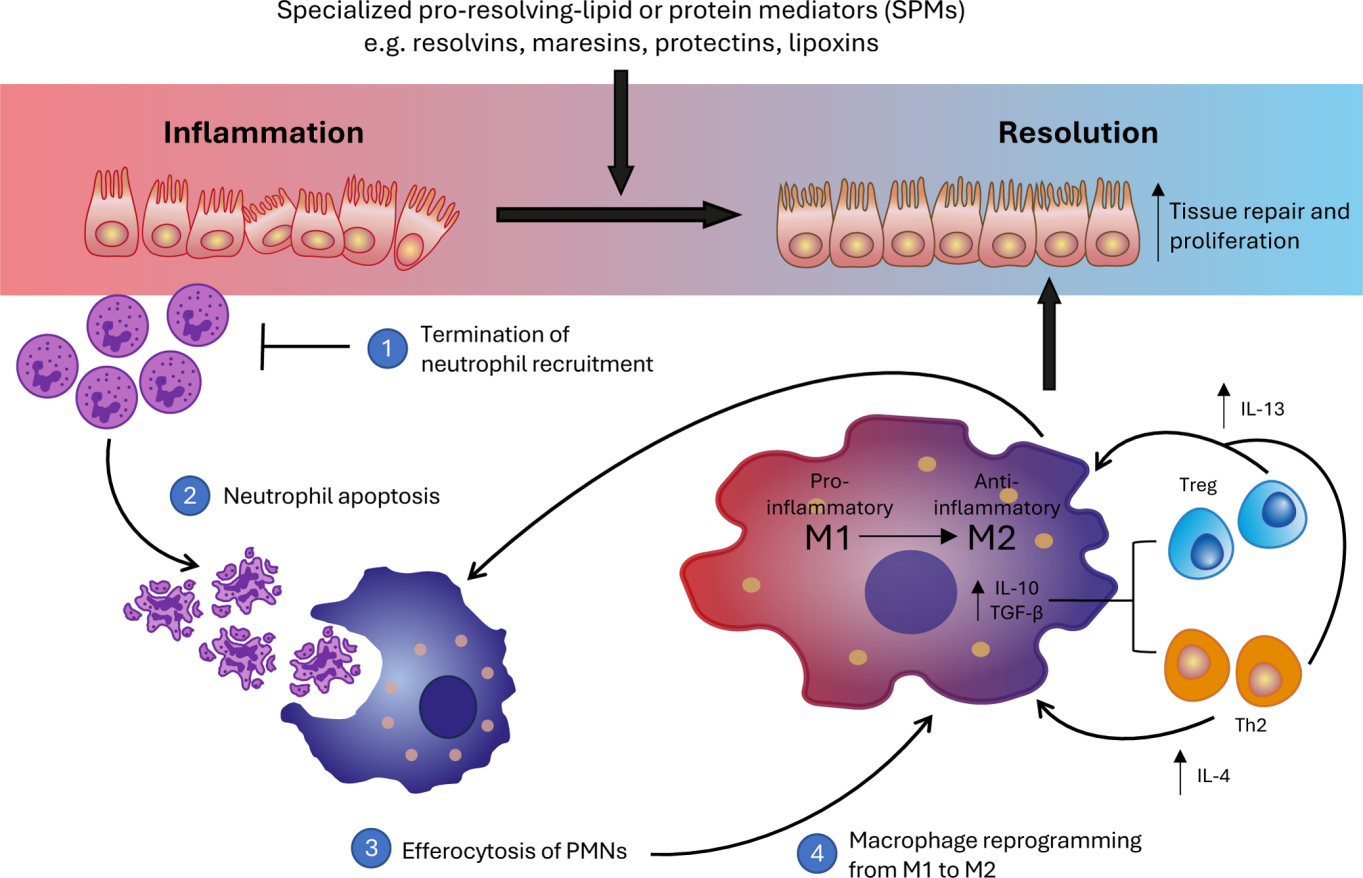
Stages of inflammation resolution. Specialized pro-resolving lipid or protein mediators (SPMs) are key mediators in controlling the cessation of pro-inflammatory pathways and initiation of tissue repair. Inflammation resolution begins with (1) termination of neutrophil recruitment followed by (2) neutrophil apoptosis. Then, macrophages clear the apoptotic PMNs through efferocytosis (3), which promotes (4) macrophage reprogramming from the proinflammatory M1 to the anti-inflammatory M2 state. M2 macrophages further support (3) and increase recruitment and differentiation of Tregs and Th2 cells, which in turn promote further transition of macrophages from M1 to M2, creating a positive feedback loop that supports tissue repair and proliferation.

In addition to myeloid cells, T cells play crucial roles in initiating and regulating inflammation resolution. T regulatory cells (Tregs) exhibit complex roles, both in controlling excessive inflammation during acute inflammation or injury and promoting tissue repair through engagement with nonimmune cells.^43,44^ For example, Tregs can stimulate the proliferation of epithelial cells within the lungs,^45,46^ skin,^47^ muscles,^48,49^ and intestines^50,51^ through the secretion of Areg and keratinocyte growth factor (KGF), both well-characterized mediators of tissue repair.^52^ Tregs have also been shown to promote the proliferation and differentiation of stem cells during skin,^53,54^ cardiac,^55^ liver,^56^ and intestinal^50,57,58^ injury and inflammation. In addition to Tregs, T-helper 2 (Th2) cells also play an important role in tissue repair and regeneration. In response to allergic inflammation or tissue damage caused by parasitic infections, Th2 cells release key tissue repair cytokines, IL-4 and IL-13,^59,60^ which promote the switching of macrophages from M1 to M2, epithelial cell proliferation and the production of extracellular matrix,^61,62^ an important component of the tissue repair process. As resident microbes have been shown to shape both lymphocyte and myeloid cell differentiation and responses, it stands to reason that the microbiome composition could modulate inflammation resolution.

## The Microbiome Shapes the Resolution of Sterile Inflammation

Within the context of sterile inflammation, or inflammation in the absence of infection, the gut microbiota has long been known to play crucial roles in maintaining and restoring tissue integrity both within and outside the gut. Several early studies in GF and antibiotic-treated animals observed distinct impairments in liver regeneration and wound healing when compared to wild type.^63,64^ Indeed, it was later shown in the dextran sodium sulfate (DSS)-induced colitis model that although GF mice exhibited decreased colonic inflammation, they suffered from exacerbated epithelial injury, hemorrhage, and increased mortality, indicating that microbiota-derived signals were required for the maintenance of a robust gut epithelial barrier.^65^ In other examples, orally administered probiotic or commensal strains of *E. coli* or *Veillonella parvula* in a mouse model of experimental autoimmune encephalomyelitis show decreased pro-inflammatory cytokines, increased IL-10 secretion from CD4+ T cells, repaired intestinal epithelial barrier dysfunction, and lower neuroinflammation (Figure 3).^66,67^ Oral administration of *V. parvula* also led to a greater resolution of inflammation in intradermal skin wounds (Figure 3). Gut-trained, commensal-specific Tregs have also been shown to migrate from the gut to injury sites, promoting tissue repair at acute sterile nonmucosal injury sites such as within skeletal muscle and liver (Figure 3).^58^ These T cells limit IL-17A production, which enables the proliferation and differentiation of stem cells, aiding injury recovery (Figure 3).^58^

**Figure 3:**
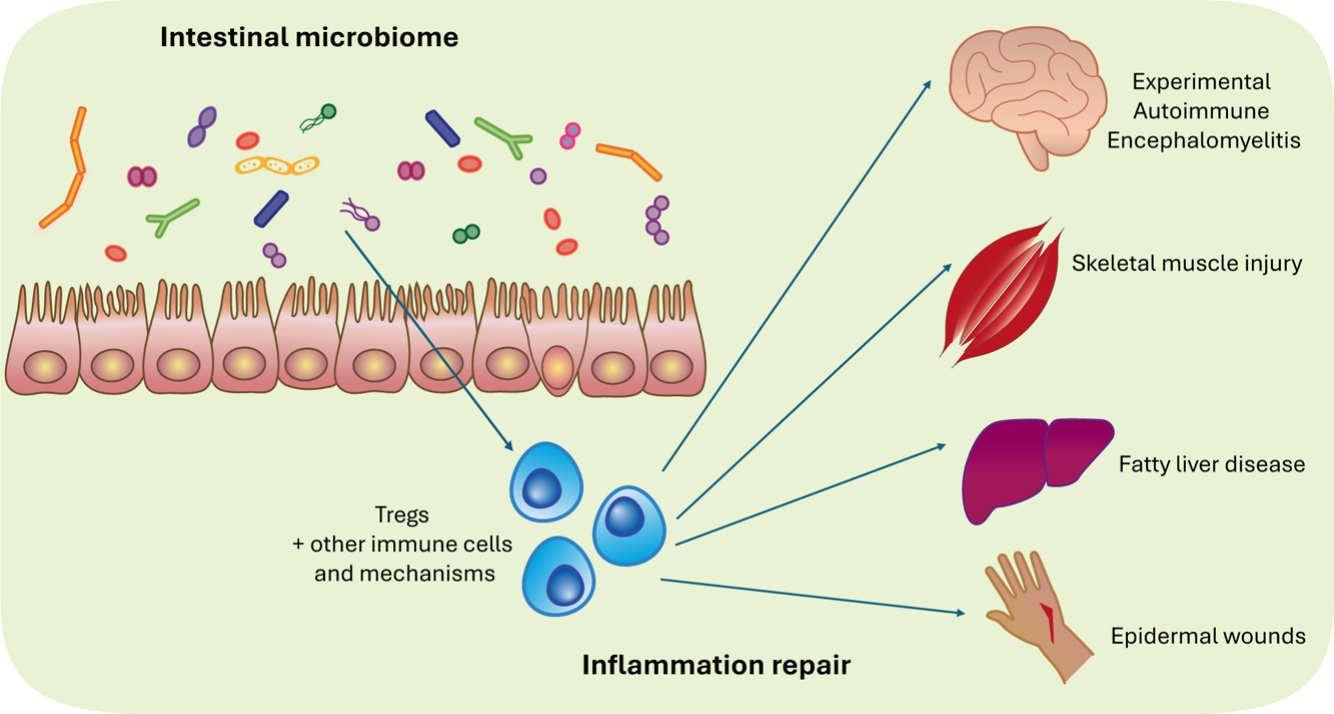
The intestinal microbiome can shape inflammation resolution at distal sites. The intestinal microbiome has been linked to both inflammation and wound repair in distal organs. Though anti-inflammatory and pro-resolving programs of commensal-specific Tregs have been shown to contribute to dampening inflammation, the mechanisms and characteristics of the commensal microbes and gut-trained immune cells that shape systemic inflammation resolution remain poorly defined.

More recently, direct mechanistic links have connected microbiome-derived signals and specific microbes with local epithelial proliferation, repair, and restoration of homeostasis. For example, microbiota-induced production of IL-22 by immune cells has been shown to mediate epithelial regeneration in numerous *in vitro* and *in vivo* studies.^57,68,69^ In another example, after administration of mechanical wounds to the distal colon of mice, the commensal bacterium *Akkermansia muciniphila* was shown to preferentially colonize damaged epithelium, which promoted proliferation and migration of adjacent enterocytes, enhancing wound repair through FPR1 and intestinal epithelial-cell-specific NOX1-dependent redox signaling (Figure 4).^70^ Another common commensal species, *Lactobacillus rhamnosus* GG, was also found to support wound repair and intestinal epithelial proliferation via increased leptin expression in the context of postradiologic intestinal injury (Figure 4).^71^ Additionally, *Lactiplantibacillus plantarum* has also been studied for its re-epithelization properties in the epidermis in which topical application resulted in accelerated wound healing in several inflammatory settings, including oxidative-related damage, excision wounds, *S. aureus* wound infections,^72^ and in abrasions of immunocompromised mice (Figure 4).^73^ Additionally, metabolites from the intestinal microbiota can also alter epithelial proliferation in response to inflammation. In several different inflammatory settings like graft-versus-host disease (GVHD),^74^ type 2 diabetes mellitus,^75^ enteric infection, and steatohepatitis,^76^ microbiota-derived butyrate improves intestinal epithelial barrier integrity, upregulates efferocytosis, and dampens systemic inflammation (Figure 4).^77^

**Figure 4:**
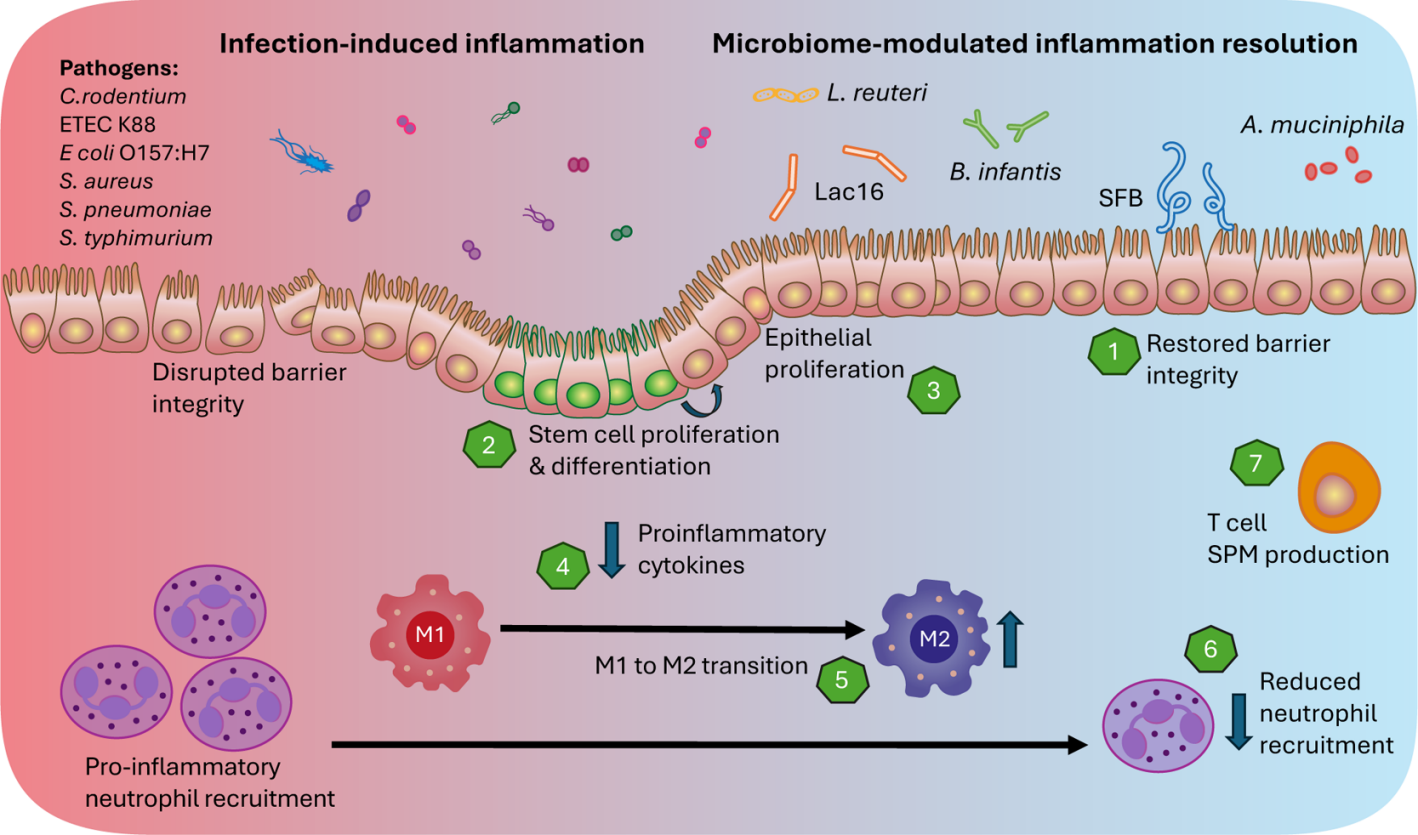
Pathogens and the interplay of commensal microbes in local resolution of infection-induced inflammation. Several commensal bacteria that reside in the intestines have been linked to local mechanisms of inflammation resolution during infection, such as (1) restoration of epithelial barrier integrity. *L. reuteri* and Lac16 stimulate (2) LgrS+ stem epithelial proliferation and differentiation, as well as (3) epithelial proliferation via upregulation of the Wnt/p-catenin signaling pathway in response to *C. rodentium*, ETEC K88, enterohemorragic *E. coli*, or MRSA infection in mice. During infection, *L. reuteri* and Lac16 have been shown to reduce (4) proinflammatory cytokine production. *B. infantis* reduces the secretion of the macrophage inflammatory proteins MIP-1a and MIP-1ß, allowing for the transition of (5) pro-inflammatory M1 macrophages to pro-resolving M2 macrophages. (6) SFB slows down neutrophil recruitment, increasing the opportunity for pro-resolving cells to clear remaining neutrophils and other apoptotic cells. Many unanswered questions remain regarding the involvement of the microbiome in local inflammation resolution, particularly the influence of (7) adaptive immune cells.

Many commensal species have also been shown to play a complex role in influencing the innate immune system, particularly keratinocyte, neutrophil, and macrophage activation, during the healing process. For example, skin commensals can stimulate IL-1β production from macrophages and keratinocytes to activate IL-1βR-88 signaling in keratinocytes, promoting hair follicle regeneration and accelerated skin re-epithelialization.^78^ *Staphylococcus epidermis*, a common skin commensal, can produce metabolites that act as a β2-adrenergic receptor antagonists on keratinocytes, which prevents the inhibition of cell motility signaling, allowing for remodeling of epithelial cells within the wound site.^79^ *Staphylococci*, *Corynebacteria* sp., and *Micrococcus luteus* trigger both *Cxcl10* gene and CXCL10 protein expression by neutrophils, recruiting plasmacytoid dendritic cells (pDCs). CXCL10 also acts as an antimicrobial protein, targeting microbes within skin wounds, forming CXCL10-bacterial DNA complexes that activate pDCs to produce type I interferons. These induce skin inflammation and wound repair via macrophages and fibroblasts.^80^

In addition to the importance at the time of active wound repair, the presence of commensal microbes in early life has also been shown to be critical for epithelial cell recovery later in life. Specific-pathogen free (SPF) mice, compared to GF mice later colonized with SPF microbiota, exhibit highly expressed erythroid differentiation regulator-1 (Erdr1) in leucine-rich repeat-containing G protein-coupled receptor 5 (Lgr5) stem cells and transit amplifying cells, which differentiate into all epithelial lineages.^81^ Erdr1 induces Wnt signaling in intestinal epithelial cells and Lgr5+ stem cell expansion, leading to an acceleration of wound repair both *in vitro* and in several intestinal inflammation mouse models, including radiation-induced injury and DSS-induced colitis.^81^ Together, these studies demonstrate that the microbiota play diverse and complex roles in modulating sterile inflammation and its resolution in local and distal sites.

## The Microbiome Can Influence Infection-Induced Inflammation Resolution

Despite numerous findings supporting the notion that microbiome composition modulates sterile inflammation resolution, the microbiome’s role in postinfection resolution remains understudied. However, few studies have defined some connections between the commensal microbiota and inflammation resolution in the aftermath of infection. For example, several commensals, particularly *Lactobacillus reuteri*, within the intestinal microbiota have been shown to play a crucial role in amplifying epithelial cell proliferation and maintaining barrier integrity, thereby aiding in the resolution of inflammation postinfection. Following *Citrobacter rodentium* infection in mice, *L. reuteri* stimulates intestinal epithelial proliferation through an upregulation of R-spondin expression and activation of the Wnt/β-Catenin pathway, resulting in an increased number of Lgr5 stem cells,^82^ which are well-known players in tissue renewal after damage.^83^ This work was further supported by similar findings where, after intestinal infection with enterotoxigenic *Escherichia coli* K88 (ETEC K88), *L. reuteri* improves epithelial barrier function integrity by inhibiting enzymes known to promote epithelial permeability and disrupt tight junction arrangement, such as myosin light-chain kinase (MLCK) and Rho-associated kinase (ROCK).^84^ Interestingly, in this infection setting, *L. reuteri* was also found to significantly reduce the expression of the pro-inflammatory cytokines, IL-8, IL-6, TNF-α, and IL-17A, suggesting that *L. reuteri* is also shaping the adaptive immune response. *Lactiplantibacillus plantarum* 16 (Lac16), a common gut species, can similarly assist in inflammation resolution by promoting epithelial cell proliferation.^85^ Following enterohemorrhagic *E. coli* O157:H7 infection in *Caenorhabditis elegans*, Lac16 also promoted epithelial repair through activation of the Wnt/β-Catenin pathway, as well as inhibition of the pro-inflammatory TLR4/MyD88 signaling pathway.^86^ Lac16 has also been shown to accelerate wound closure and reduce pro-inflammatory cytokine expression in MRSA-infected wounds of diabetic rats.^87^

Beyond epithelial repair, commensals can also influence the innate and adaptive immune responses that promote the resolution of infection-associated inflammation. In an immunocompromised (Rag^–/–^) mouse model colonized with segmented filamentous bacteria (SFB), mice exhibited a significant drop in inflammatory neutrophils and an increase in pro-resolution macrophages during the resolution phase of *S. pneumoniae* infection. This resulted in less severe pathology and faster recovery from infection.^88^ Additionally, in *Salmonella*-infected mice, the commensal *Bifidobacterium infantis* 35624 has been demonstrated to promote inflammation resolution and tissue repair.^89^ *B. infantis* 35624 greatly reduced secretion of the macrophage inflammatory proteins, MIP-1α and MIP-1β, which help recruit pro-inflammatory neutrophils, macrophages, and monocytes to sites of inflammation within *Salmonella*-infected mice.^89^ Of note, this was found to inversely correlate with CD4+CD25+ T cell populations, but the potential role of these T cells in inflammation recovery was not defined.^89^ Collectively, these studies have begun to reveal some mechanisms by which the microbiome can modulate infection-induced inflammation resolution.

Additionally, commensal-induced antimicrobial peptides (AMPs) may also play a role in promoting the resolution of infection-induced inflammation. AMPs are an important arm of innate immunity to pathogens that also modulate the host immune response and promote wound repair.^90^ Recently, commensal bacteria have been shown to upregulate important AMPs, including Reg3γ,^91,92^ RELM-α^96^ and -β,^94^ and defensins.^95^ In helminth infections, the AMP RELM-α has been shown to mitigate inflammatory responses by downregulating *Il17* expression in the lungs after *Nippostrongylus brasiliensis* infection, reducing the severity of helminth-induced emphysema.^93^ Furthermore, in an infection-induced mouse colitis model, RELM-β has been shown to reduce infection severity and mucosal damage by promoting CD4+ T cell recruitment and IL-22 production to support epithelial cell proliferation.^94^ However, direct connections between commensal bacteria and inflammation resolution through the regulation of AMP production remain to be established and represent a potential avenue for further study. Together, these studies have begun to reveal some potential mechanisms by which the microbiome can modulate the infection-induced inflammation resolution.

## The Other Edge of the Sword: The Microbiome Can Also Impair Inflammation Resolution

While certain commensal bacteria have been shown to have beneficial effects on chronic inflammation, resident microbes have also been shown to hinder or prevent inflammation resolution. Despite previous findings showing that several species of the *Lactobacillus* genus accelerate inflammation resolution,^82,96,97^ one study found that in an epidermal wound model, *Lactobacillus acidophilus* can result in increased accumulation of pro-inflammatory immune cells, leading to delayed re-epithelialization and poor wound healing.^97^ Another study found that *Enterobacteriaceae,* mainly *Proteus mirabilis*, contribute to intestinal inflammation postinjury by stimulating IL-1β release by monocytes in an NLRP3 inflammasome-dependent manner.^98^

The microenvironment within a wound has been shown to facilitate the preferential growth of specific commensals. These changes within the microbial community can lead to hindrance or acceleration of wound and tissue repair, depending on the conditions of the microenvironment and the characteristics of the microbes. In many settings, poor healing of skin wounds has been linked with facultative and strict anaerobes,^99–102^ particularly species of the *Enterobacter* ^103^ and *Enterococcus* ^104^ genera. This is particularly apparent in deep, unresolved diabetic wounds^99,100,105^ and pressure ulcers.^106^ Interestingly, poor inflammation resolution has also been associated with a stable wound microbiota over time,^107,108^ suggesting that the dynamic microbial changes within the wound are essential for regulating repair. In support of this, after antibiotic treatment, the microbiome composition of murine skin lesions exhibited markedly decreased populations of several commensal *Staphylococcus* species, which was correlated with decreased expression of Reg3γ and IL-17 and poorer re-epithelialization.^109^ A reintroduction of these commensal species within the microenvironment rescued the healing process.^109^

Commensal microbes at other mucosal sites have also been found to drive the development of inflammatory diseases. For example, the excessive proliferation of *Gardnerella vaginalis*, a common member of the vaginal microbiome, is associated with bacterial vaginosis. Overgrowth of *G. vaginalis* results in epithelial barrier disruption and increased pro-inflammatory immune cell activation. The soluble products from *G. vaginalis* cultures also inhibit the healing of epithelial cell monolayers *in vitro*, suggesting that this bacterial species prevents effective immune responses and wound repair, leading to chronic, unresolved inflammation.^110^ The progression of chronic obstructive pulmonary disease (COPD) has also been linked to commensal bacteria within the local microenvironment. In particular, mice with secretory immunoglobulin A (SigA) deficiency, a common phenotype shared by COPD patients, exhibit spontaneous COPD-like pathology only in the presence of a replete microbiota, and this pathology is absent in GF mice. This illustrates that the resident lung microbiota can lead to persistent activation of the innate immune system in SigA deficient mice.^111^ Ultimately, these findings underscore the importance of understanding the complex mechanisms by which the microbiome impacts immune responses, inflammation, and inflammation resolution. This understanding would provide a platform for the rational design of improved strategies for preventing or treating acute and chronic inflammation and associated tissue damage.

## Current Strategies for Pro-Resolving Therapeutics

Anti-inflammatory and immunosuppressant therapeutics have long been the focus of inflammation research and are widely used clinically to treat both acute and chronic inflammation. However, despite the widespread use of these therapies for treating inflammatory conditions, they often exhibit incomplete success. For example, classic anti-inflammatories and immunosuppressants are largely considered palliative for the treatment of inflammatory bowel disease (IBD) as they fail to yield sufficient mucosal healing.^112^ Furthermore, biologic therapies, such as TNF-inhibitors, only achieve about 50% clinical remission in severe IBD cases and often wane in efficacy over time.^112,113^ Steroids are another essential tool in the anti-inflammatory arsenal that distinctly exhibit both anti-inflammatory and pro-resolving properties, but they are notorious for adverse effects.^114–116^ Therefore, there remains an unmet need for effective therapeutic interventions for acute and chronic inflammation.

Due to the shortcomings of current anti-inflammatory drugs, immunosuppressants, and steroids, there has been increasing interest in developing pro-resolving therapeutics.^117–119^ Instead of suppressing the overall immune response, these pro-resolving drugs aim to clear inflammatory signals and promote a return to homeostasis. Some of the current strategies involve combining anti-inflammatory drugs with treatments that promote inflammation resolution. For example, roscovitine or AT7519, which can enhance neutrophil apoptosis, can be paired with steroids or lipid mediators that promote macrophage efferocytosis to clear the apoptotic cells.^120,121^ These drugs may provide potential for treating acute inflammatory diseases where prolonged inflammation can lead to long-term consequences, such as bleomycin-induced lung inflammation,^120,122,123^ as well as diseases associated with excessive accumulation of neutrophils, such as inflammatory bowel disease and rheumatoid arthritis (RA).^124^ This combination therapy shown some success in a pneumococcal meningitis mouse model, promoting greater tissue repair and infection recovery.^125^ Similarly, Ap1189, a synthetic biased melanocortin receptor agonist that can reduce the release of pro-inflammatory cytokines and promote efferocytosis of apoptotic neutrophils, has been shown to accelerate recovery in severe COVID-19 patients with minimal adverse effects.^126–128^ It has also been shown to reduce pathology and promote recovery in a mouse model of arthritis, and clinical trials are now underway for its use in RA patients.^126^ Another possible avenue is the development of therapeutic mimetics of lipid mediators, such as PGJ_2_ and RvD1, as these have been shown to initiate innate-immune inflammation resolution.^129,130^ Annexin-A1 mimetic peptides have also shown promising results as an inflammation-resolving therapeutic. Specifically, in a rat myocardial infarction model, RTP-026 has been shown to reduce recruitment of neutrophils and monocyte activation, and this drug is currently in a phase 2a clinical trial for acute myocardial infarction.^126,131^

However, it is important to note that most research examining potential pro-resolving therapeutics has primarily focused on resolving acute inflammation, which lacks the same complexity and challenges as chronic inflammation. Thus, more studies on pro-resolving candidates in animal models that better recapitulate chronic inflammation pathology are needed. Additionally, there has been a concern that accelerated inflammation resolution could allow for opportunistic secondary infections if innate antimicrobial responses are dampened.^117,132^ However, there is evidence to suggest that SPMs can augment cellular antimicrobial function by increasing neutrophil and macrophage bacterial phagocytosis, allowing for inflammation resolution that does not compromise host defense.^133^

Given the close interactions between the microbiome and the immune system and increasing evidence of their role in inflammation resolution, further investigation of the microbiota’s ability to produce or regulate the production of SPMs could uncover new therapeutic avenues to control infection-associated inflammation and prevent or treat associated chronic inflammatory diseases.

## Conclusions and Future Prospects

Further functional microbiome research will deepen our understanding of microbiota-mediated modulation of host immune responses and host physiology, including the role of resident microbes in postinfection inflammation resolution. Integrating both bioinformatics approaches, such as functional metagenomics, and interdisciplinary experimental methods will help elucidate the causal relationships and the underlying mechanisms behind this host-microbiota-immune crosstalk. However, an experimental investigation of these relationships will require additional and better animal models of infection-induced inflammation and autoimmunity. These models will also benefit from compatibility with simplified microbial consortia and/or the introduction of more diverse and complete microbial ecosystems such as those achieved with wild-derived microbiota transplantation. In addition, as most studies introduce or perturb the microbiota prior to infection, more detailed characterizations of the contribution of commensal microbes at different stages of infection and after pathogen clearance will be essential. Beyond the pathogens known to trigger sustained host pathology and chronic inflammation, there are likely additional infection-disease connections that have yet to be identified. These new avenues for research in infection and inflammation have the potential to yield crucial insights into the etiology of numerous diseases.

In conclusion, the intricate relationship between the resident gut microbiota and host health extends far beyond simple competition with pathogens, encompassing critical roles in both preventing infection and actively resolving the subsequent inflammation and tissue damage. While the protective mechanisms of commensal microbes in warding off invaders have been recognized for over a century, recent research illuminates their profound influence on host immune responses, epithelial repair, and the active process of inflammation resolution. From specific strains like *E. coli* Nissle and *L. reuteri* promoting barrier integrity and tissue regeneration to the broader impact of microbial metabolites like butyrate on dampening inflammation and enhancing efferocytosis, it is clear that the microbiome is an indispensable partner in maintaining host homeostasis postinfection. A deeper understanding of these complex cellular and molecular interactions will pave the way for developing novel therapeutics aimed at mitigating infection-induced pathologies and inflammatory diseases.
